# Long‐term observation of the frequency of secondary colorectal cancer and other malignancies in tyrosine kinase inhibitor treated chronic myeloid leukemia patients and controls

**DOI:** 10.1002/jha2.502

**Published:** 2022-06-07

**Authors:** Nina Winkelmann, Michaela Schwarz, Bert Hildebrandt, Oliver Henke, Lars Bullinger, Il‐Kang Na, Sebastian Stintzing, Philipp le Coutre

**Affiliations:** ^1^ Department of Hematology, Oncology, and Cancer Immunology, Campus Charité Mitte Charité‐Universitätsmedizin Berlin Berlin Germany; ^2^ Department of Hematology, Oncology, and Cancer Immunology, Campus Virchow‐Klinikum Charité‐ Universitätsmedizin Berlin Berlin Germany

**Keywords:** chronic myeloid leukemia, colorectal cancer, secondary malignancy

## Abstract

In this analysis, we examined the risk of secondary malignancies for tyrosine kinase inhibitor (TKI) therapy in chronic myeloid leukemia (CML) patients. We also collected data on specific risk factors for colorectal cancer. Ninety‐one patients with CML and 76 controls were included and in total 4 (4.4%) secondary malignancies were found in patients and 8 (10.5%) in controls. The risk for secondary malignancies was not significantly elevated for CML patients (*p* = 0.141). Two (2.2%) CML patients developed colorectal cancer compared to 4 (5.3%) in the reference group. A higher risk for CML patients for colorectal cancer could not be found (*p* = 0.414).

## 1 INTRODUCTION

In this study, we examined the risk of colorectal and other secondary cancers for any tyrosine kinase inhibitor (TKI) therapy in BCR::ABL1 chronic myeloid leukemia (CML) patients using a comparative study design in 91 patients with CML and 76 controls and collected data on specific risk factors.

Since the introduction of therapeutic TKIs, the life expectancy of CML patients is now comparable to the general population [1]. Therefore, the increased prevalence of CML shifted the clinical interest to the impact of long‐term toxicities and secondary malignancies [2, 3].

Roy et al. first described in a cohort of 189 patients 6 (3.2%) secondary malignancies (carcinomas of prostate, urinary bladder, and colon) after a median follow‐up of 5 years and of TKI exposition of 2.6 years but a higher overall incidence could not be detected [4]. In a larger cohort of 1445 patients with chronic myeloproliferative neoplasms after a median follow‐up of 39 months, Verma et al. found a standardized incidence rate (SIR) of 0.6 that was lower than expected in the normal population. In this investigation, malignant melanoma, endocrine tumors, and renal cell carcinoma as individual events were seen more frequently [5]. In a Swedish study, a total of 868 patient was analyzed with a median follow‐up of 3.7 years. Forty‐nine patients with secondary neoplasms (primarily gastrointestinal and head and neck cancers) were identified accounting for an elevated SIR compared to the normal population [6]. In the German CML IV study cohort of 1525 patients, 64 individuals with secondary malignancies were identified (prostate, colon, lung, breast, NHL, and others), but only Non Hodgkin's lymphoma (NHL) had a higher SIR [7]. The most recent study examined SEERS data of 9200 CML patients that showed a 30% elevated rate of cancer in CML patients compared to the SIR of the normal population [8].

So far, the present data on secondary malignancies are not conclusive as (i) all previous studies were lacking a control group, (ii) previous studies were not focusing on one individual cancer but were investigating the occurrence of secondary neoplasms as one event, and (iii) follow‐up in previous studies was short.

With a median follow‐up of 89 (IQR 101) months, our study provides a long observation period. Data were captured between 01.01.2015 and 31.12.2017 and each patient household was provided with a questionnaire and an informed‐consent form. Eligibility criteria were diagnosis of BCR::ABL1 positive CML in (1) chronic phase, present, or previous TKI therapy and age ≥ 18 years. Due to the relatively small cohort, no prospective statistical plan was implemented and a retrospective analysis was preferred.

The control group consisted in individuals sharing the same household and was primarily spouses, partners and less often cases children, siblings or parents of patients. Eligibility criteria for controls were no diagnosis of CML, no current or past therapy with a TKI, and age ≥ 18 years.

The questionnaire consisted of general questions that included BMI, gender, age, relationship to patient, preexisting comorbidities, and current medication. CML‐related questions included duration of CML and TKI therapy, stage of disease, and toxicities. Questions related to the risk of colorectal neoplasia were derived from the recommendations of the German Society of Hematology and Oncology [9]. Secondary cancers other than of colorectal origins were detected by an open question. Statistical analysis was performed using *t*‐test, chi‐square test, and Fisher test. This study was conducted in accordance with the Declaration of Helsinki.

Study documents were distributed to CML patients of our center and 94 questionnaires were returned. Of these, 3 (3.2%) were not appropriate for analysis. Thus, the analyses were performed on 91 CML patients and 76 controls (Table 1).

**TABLE 1:** Characteristics of CML patients (*n* = 91) and controls (*n* = 76)

	**CML patients**				**Controls**				
	**Total**	**%**	**Mean**	**SD**	**Total**	**%**	**Mean**	**SD**	**Fisher test (*p*)**
All	91				76				
Age (years)			58.0	±14.3			56.5	±15.1	
Gender									0.089
M	53	58.2**%**			34	44.7**%**			
F	38	41.8**%**			42	55.3**%**			
BMI			27.3	±5.5			25.9	±4.7	
Comorbidities									
Hypertension	46	50.6**%**			29	38.2**%**			0.120
Diabetes mellitus	12	13.2**%**			9	11.8**%**			0.820
Atherosclerosis	11	12.1**%**			0	0.0**%**			0.001
Cardiac	12	13.2**%**			5	6.6**%**			0.202
Cancer	15	16.5**%**			8	10.5**%**			0.368
Tobacco abuse	14	15.6**%**			16	21.1**%**			0.420
Alcohol abuse	4	4.4**%**			9 (75)	12.0**%**			0.085
Dietary risk factors	46	50.6**%**			27 (73)	37.00**%**			0.114
Completed questionnaire	90	98.9**%**							
Age at early diagnosis (years)	48.6			±12.8					
Duration of CML (months)	88.5 (Median)			101 (IQR)					
Duration of TKI exposure (months)	82.0 (Median)			95(IQR)					
Pre‐TKI therapy	35	38.9**%**							
IFN‐α	6	6.7**%**							
HU	16	17.8**%**							
IFN‐α + HU	13	14.4**%**							
Previous TKI									
Imatinib	63	70.0**%**							
Nilotinib	52	57.8**%**							
Dasatinib	25	27.8**%**							
Bosutinib	4	4.4**%**							
Ponatinib	13	14.4**%**							
Line of therapy									
First line	46	51.1**%**							
Second line	25	27.8**%**							
Third line and beyond	19	21.1**%**							

Abbreviations: BMI, body mass index; Cardiac, coronary heart disease and infarction; F, female; DM, diabetes mellitus; M, male;SD, standard deviation.

No significant differences between CML patients and controls were found for median age (57.96 ± 14.28 vs. 56.48 ± 15.09 years), male to female ratio [53 (58%) vs. 38 (42%) and 34 (45%) vs. 42 (55%)] or BMI (27.27 ± 5.53 vs. 25.92 ± 4.72) (Table 1).

Also, no significant differences were found between the CML and control cohorts for comorbidities such as arterial hypertension [*n* = 46(51%) vs. *n* = 29(38%)], diabetes mellitus [*n* = 12(13%) vs. *n* = 9(12%)], coronary heart disease or infarctation [*n* = 12(13%) vs. *n* = 5(7%)] or preexisting cancer [*n* = 15(16%) vs. *n* = 8(11%)]. Only atherosclerosis occurred more frequently in the CML group [*n* = 11(12%) vs. *n* = 0(0%), *p* = 0.001] (Table 1).

The median age at diagnosis of CML was 49 (±12,8) years. The median duration since first diagnosis of CML was 89 (IQR 101) months. The median duration of TKI therapy was 82 (IQR 95) months (Table 1).

Of 90 CML patients with a completed survey 6 (6,7%) previously received IFN‐α, 16 (17.8%) hydroxyurea and 13 patients (14.4%) both (Table 1).

Altogether imatinib was taken by 63 (70%), nilotinib by 52 (57.8%), dasatinib by 25 (27.8%), bosutinib by 4 (4.4%), and ponatinib by 13 (14.4%) of all patients. As first line TKI therapy imatinib was used in 58 (64.4%), nilotinib in 22 (24.2%), dasatinib in 5 (5.6%), bosutinib in 2 (2.2%), and ponatinib in 3 (3.3%) patients. Forty‐six (51.1%) of patients were in first line, 25 (27.8%) in second line, and 19 (21.1%) in third line or beyond.

Of 91 CML patients, two (2.2%) and four (5.3%) out of 76 individuals in the control group developed colorectal cancer. There was no statistically elevated rate of colorectal cancer in the CML group (*p* = 0.414) (Table 2). No statistically different risk profiles were detected in CML patient versus controls (Table 2). Exclusively stool irregularity was present more frequently in 35 (38.9%) of CML patients as compared to 10 (14.5%) controls (*p* < 0.003) (Table 2).

**TABLE 2:** Risk factors of colorectal cancer CML patients versus control group

	**CML**			**Control**			***p*‐Value**	
	**Total**	**Percent (%)**	**Mean (SD)**	**Total**	**Percentent (%)**	**Mean (SD)**	**Fisher test**	**Chi^2^ test**
Number	91			76				
Age			57.96 (14.28)			56.5 (15.09)		
Gender								
Male	53	58.2		34	44.7			0.082
Female	38	41.8		42	55.3			
BMI			27.27 (5.53)			25.9 (4.72)		
Adipositas	20 (90)	22.2		13 (73)	17.8		0.559	0.486
Family History	3 (89)	3.5		6 (74)	8.1		0.302	0.187
Benign Lesion	10 (89)	11.5		3 (75)	4.0		0.145	0.087
FAP	0	0.0		0 (75)	0.0		1	
CID	2 (88)	2.3		1 (74)	1.4		1	0.665
Colitis Ulcerosa	2 (88)	2.3		0 (74)	0.0		0.501	
Crohn's disease	0 (88)			1 (74)	1.4		0.457	
Tobacco								
Never	45 (90)	50		35	46.1		0.642	0.612
Ex‐smoker	31 (90)	34.4		25	32.9		0.87	0.833
Smoker	14 (90)	15.6		16	21.1		0.42	0.359
Alcohol								
Never	32	35.2		21 (75)	28.0		0.403	0.324
Occasional	55	60.4		45(75)	60.0		1	0.954
Frequent	4	4.4		9 (75)	12.0		0.085	0.070
High risk diet	46	51.7		27 (73)	37.0		0.114	0.82
Prophylaxis	60	68.5		49	64		0.871	0.844
Coloscopy	54	59.3		39	51.3			
Hämoccult	41	45.1		33	43.4			
Unregular colon passage	35 (90)	38.9		10	13.2		0.0002	0.000203
Abominal pain	23 (89)	25.8		10 (72)	13.9		0.078	0.062
CRC	2	2.2		4	5.3		0.414	0.296

Abbreviations: BMI, body mass index; CID, chronic inflammatory disease; CRC, colorectal cancer; FAP, familial adenomatous polyposis.

Both CML patients with secondary colorectal cancer were females with a median age of 75 ± 2.83 years and a median BMI of 25.61 ± 2.45. In these individuals, risk factors associated with colorectal cancer were not significantly different when compared to the rest of CML patients (Table S1).

In addition to colorectal cancers, six additional malignancies were found, two of them occurred in the CML group and four in the control group (prostate cancer, basal cell carcinoma, breast cancer, and malignant melanoma) (Table S2).

Of the four CML patients with secondary cancers, two exclusively received imatinib and two received nilotinib, dasatinib, and ponatinib as well. The median age of these four patients at diagnosis was 59.5 years (IQR 7) compared to 48 (IQR 20) in CML patients without a secondary cancer. The median duration of CML of these four patients was 167.5 months (IQR 12) as compared to 87 months (IQR 101) in CML patients without a secondary cancer.

Finally, the mean duration of TKI therapy was 134 months (44 IQR) in patients with secondary cancer as compared to 81 months (IQR 92) in CML patients without secondary cancer (Table S3).

In our study group, we observed a wide range for disease duration (range: 1–299 months) and TKI treatment (range: 1–179 months). Despite this long retrospective availability of data, we may have missed secondary cancers occurring after 2017 when data collection was stopped after two years.

The rate of 4.2% secondary cancers in the German CML study group in a cohort of 1525 patients is matching our findings (4.4%) and is interesting as these patients exclusively were in first line but were followed up shorter (67 months) than our patients (89 months) [7, 10].

In this study, we actively asked for colorectal risk factors as well as previously performed screening endoscopies. We therefore reached a higher level of accuracy with respect to colon cancer when compared to other studies that also looked for secondary cancers [7].

The median age of our CML cohort (49 years) was lower than in the Swedish (60 years) and German (52 years) studies [6, 7]. This could potentially be explained by the monocentric nature of our cohort as especially younger patients are frequently referred to our academic center.

The median duration of disease was only provided in the German study and was shorter than in our study (67.5 vs. 89 months) [7].

Our survey also had a special focus on various risk factors of colorectal cancer. These were primarily age, BMI, tobacco and/or alcohol abuse, and comorbidities. In our study, we did not observe and differential distribution of risk factors when compared to controls with the only exception being gastrointestinal symptoms (Table 2).

We observed more often abdominal pain (25.8% vs. 13.9%, *p* = 0.062) and irregular defecation (38.9% vs. 14.5%, 0.0002) between CML patients and controls (Table 2).

This risk factor shows a statistically significant difference between the two groups but may be well explained by the toxicity profile of TKIs that contains abdominal discomfort [11, 12, 13, 14].

We could not detect any differential rate of colorectal cancer in CML patients when compared to controls. two (2.2%) of CML patients versus 4 (5.3%) of controls developed a colorectal cancer.

These data are reflecting the results of Miranda et al. (2016) with 6/1525 cases (0.4%) of colorectal cancer [7]. A higher risk for all gastrointestinal tumors was noted by Gunnarsson et al. (2015) with 13 GI‐tumors in 868 patients [6].

Finally, Kumar et al. (2018) found a numerically higher risk for colon cancer (0.46%) but a lower risk for rectal cancer (0.05%) in 9200 CML patients [8].

Aside from the two cases of colorectal cancer in the group of CML patients one patient with basal cell carcinoma and one with prostate cancer were identified accounting for 4 (4.4%) secondary malignancies. Compared to the altogether eight cases (10.7%) of secondary cancers in the control group, this difference is not beyond statistical significance (*p* = 0.141). As part of the institutional requirements of our center, all our data were presented to an intramural statistical analysis. The limitations of our study cohort based on a relatively small case number were acknowledged but statistical accuracy was confirmed. As part of this procedure full recognition as a medical thesis was provided.

In conclusion, our study did not demonstrate an elevated risk of secondary malignancies in TKI‐treated CML patients.

## CONFLICT Of INTEREST

The authors declare that there is no conflict of interest that could be perceived as prejudicing the impartiality of the research reported.

## FUNDING INFORMATION

This study did not receive any special funding.

## ETHICS STATEMENT

This study was part of the medical thesis of the first author. All participants of this study gave their written informed consent.

## Supporting information

Supporting Information
