# Supplementary material for: Long‐term observation of the frequency of secondary colorectal cancer and other malignancies in tyrosine kinase inhibitor treated chronic myeloid leukemia patients and controls
Source: EJHaem. 2022 Jun 7;3(3):949–53. doi: 10.1002/jha2.502 (PMC9422034; doi:10.1002/jha2.502)
Supplement: Supplementary file 1 — Supporting Information [file JHA2-3-949-s001.docx]

| **Table 3 = Supplementary table 1.** Patients with colorectal cancer. CML patients (n=2) versus controls (n=4) | | | | | |
| --- | --- | --- | --- | --- | --- |
|  | CML | |  | Controls | |
|  | total | mean (SD) |  | Total | Mean (SD) |
| total group | 90 |  |  | 76 |  |
| total | 2 |  |  | 4 |  |
| Age |  | 75 (2.83) |  |  | 73.75 (14.9) |
| Gender |  |  |  |  |  |
| Male | 0 |  |  | 3 |  |
| Female | 2 |  |  | 1 |  |
| BMI |  | 25.61 (2.45) |  |  | 24.25 (1.99) |
| Adipositas | 0 |  |  | 1 |  |
| Family History | 0 |  |  | 0 |  |
| benign lesion | 0 |  |  | 1 |  |
| FAP | 0 |  |  | 0 |  |
| CID | 0 |  |  | 0 |  |
| Colitis Ulcerosa | 0 |  |  | 0 |  |
| Crohn`s disease | 0 |  |  | 0 |  |
| Tobacco |  |  |  |  |  |
| Never | 1 |  |  | 2 |  |
| Ex-smoker | 0 |  |  | 2 |  |
| Smoker | 1 |  |  | 0 |  |
| Alcohol |  |  |  |  |  |
| Never | 2 |  |  | 2 |  |
| Occasionally | 0 |  |  | 2 |  |
| Ferquently | 0 |  |  | 0 |  |
| High risk diet | 1 |  |  | 1 |  |
| Prophylaxis | 2 |  |  | 4 |  |
| Colonoscopy | 2 |  |  | 4 |  |
| Hämoccult | 1 |  |  | 2 |  |
| Stuhlunregelmäßigkeiten | 1 |  |  | 1 |  |
| abdominelle Schmerzen | 1 |  |  | 0 |  |
| Body-Mass-Index: BMI, Raumforderung (RF), Familial adenomatous polyposis (FAP), Chronic inflammatory disease (CID) | | | | | |

| **Supplementary table 2**  All secondary malignancies CML-group (n=91) versus controls (n=75). | | | | | |
| --- | --- | --- | --- | --- | --- |
|  | CML |  | Controls |  |  |
|  | Post-TKI |  |  |  | Fisher-Test |
| Colorectal cancer | 2(3) |  | 4 |  | 0.414(0.703) |
| Basal cell carcinoma | 1(3) |  | 1 |  | 1(0.627) |
| Squamous cell carcinoma | 0(1) |  | 0 |  | 1(1) |
| Prostate cancer | 1(1) |  | 1 |  | 1(1) |
| Renal cell carcinoma | 0(2) |  | 0 |  | 1(0.502) |
| Breast Cancer | 0(3) |  | 1 |  | 0.455(0.627) |
| Malignant Melanoma | 0(1) |  | 1 |  | 0.455(1) |
| Myxofirbrosarkoma | 0(1) |  | 0 |  | 1(1) |
| Nasopharyngeal carcinoma | 0(1) |  | 0 |  | 1(1) |
| other | 0(1) |  | 0 |  | 1(1) |
| Total | 4(17) |  | 8 |  | 0.141(0.285) |
| Tyrosine Kinase Inhibitor: TKI | | | | | |

| **Supplementary table 3.** CML patients with all secondary cancer versus CML patients without secondary cancer | | | | | | | |
| --- | --- | --- | --- | --- | --- | --- | --- |
|  | Secondary cancer | | |  | No secondary cancer | | |
|  | Total | Mean (SD) | Median (IQR) |  | Total | Mean (SD) | Median (IQR) |
| Total | 4 |  |  |  | 86 |  |  |
| Family history | 0 |  |  |  | 3 |  |  |
| Age at early diagnosis (years) |  | 58.25 (3.78) |  |  |  | 48.19 (12.9) |  |
| CML duration (months) |  |  | 167.5 (129) |  |  |  | 87 (101) |
| Pre-TKI therapy | 3 |  |  |  | 32 |  |  |
| IFN α | 2 |  |  |  | 17 |  |  |
| Imatinib | 4 |  |  |  | 59 |  |  |
| Nilotinib | 2 |  |  |  | 50 |  |  |
| Dasatinib | 2 |  |  |  | 23 |  |  |
| Bosutinib | 0 |  |  |  | 4 |  |  |
| Ponatinib | 2 |  |  |  | 11 |  |  |
| TKI duration of exposure (months) |  |  | 134 (44) |  |  |  | 81 (92) |
| Early diagnosis until CRC (months) |  |  | 135 (153) |  |  |  |  |
| TKI start until CRC (months) |  |  | 107 (53) |  |  |  |  |
|  | | | | | | | |
